# A small microring array that performs large complex-valued matrix-vector multiplication

**DOI:** 10.1007/s12200-022-00009-4

**Published:** 2022-04-28

**Authors:** Junwei Cheng, Yuhe Zhao, Wenkai Zhang, Hailong Zhou, Dongmei Huang, Qing Zhu, Yuhao Guo, Bo Xu, Jianji Dong, Xinliang Zhang

**Affiliations:** 1 Wuhan National Laboratory for Optoelectronics, School of Optical and Electronic Information, Huazhong University of Science and Technology, Wuhan, 430074 China; 2 Photonics Research Centre, Department of Electronic and Information Engineering, The Hong Kong Polytechnic University, Hong Kong, 999077 China; 3 The Hong Kong Polytechnic University Shenzhen Research Institute, Shenzhen, 518057 China; 4 Photonics Research Centre, Department of Electrical Engineering, The Hong Kong Polytechnic University, Hong Kong, 999077 China; 5 Institute of Strategic Research, Huawei Technologies, Shenzhen, 518129 China

**Keywords:** Photonic matrix–vector multiplication, Complex-valued computing, Microring array, Signal/image processing

## Abstract

As an important computing operation, photonic matrix–vector multiplication is widely used in photonic neutral networks and signal processing. However, conventional incoherent matrix–vector multiplication focuses on real-valued operations, which cannot work well in complex-valued neural networks and discrete Fourier transform. In this paper, we propose a systematic solution to extend the matrix computation of microring arrays from the real-valued field to the complex-valued field, and from small-scale (i.e., 4 × 4) to large-scale matrix computation (i.e., 16 × 16). Combining matrix decomposition and matrix partition, our photonic complex matrix–vector multiplier chip can support arbitrary large-scale and complex-valued matrix computation. We further demonstrate Walsh-Hardmard transform, discrete cosine transform, discrete Fourier transform, and image convolutional processing. Our scheme provides a path towards breaking the limits of complex-valued computing accelerator in conventional incoherent optical architecture. More importantly, our results reveal that an integrated photonic platform is of huge potential for large-scale, complex-valued, artificial intelligence computing and signal processing.

## Introduction

With the rapid advancement of technology in recent decades, there is a growing demand for large-capacity, high-speed computing over traditional computing. This is especially seen in the field of convolutional processing, a computationally intensive operation in electronics that occupies over 80% of the total processing time for image processing [1–3]. Optical computing has the ability of parallel processing with wavelength division multiplexing (WDM) due to its intrinsic high speed and low power consumption, thus has been proposed as a promising candidate for mass data processing [4]. Matrix multiplication is the kernel and most common operation in artificial intelligence (AI). It is widely used in artificial neutral networks (ANNs), which have been universally applied in signal processing, imaging recognition, voice recognition, real-time video analysis, and autonomous driving [5, 6]. The optical neural networks (ONNs) can improve the computation speed by several orders of magnitude. For example, a photonic convolutional accelerator comprised of soliton microcombs could carry out up to 10 trillion operations per second [7]. In addition, phase-change material (PCM) has been employed in non-volatile memory storage in optical computing to reduce the energy consumption of optical-electrical conversion during weight data refreshing [8–11]. Recently, an integrated photonic hardware accelerator has successfully executed ${10}^{12}$ multiply-accumulate operations per second by combining phase-change-material memory and soliton microcombs [9].

A copious amount of research has been conducted in optical matrix computing using spatial light modulators [12, 13], electro-optic modulations [14–16], direct driven LED arrays [17], acousto-optic Bragg cells [18–20], and photorefractive medias [21–23]. Although spatial light modulators and other spatial elements are easily programmable, these methods are in general bulky, complex, and power-consuming. With the advancement of integrated photonics technology and hardware implementation of nanophotonic processors, integrated photonic platforms have shown huge potential for high-performance computing. At present, most existing neural networks are based solely on real-valued algorithms, but complex-valued algorithms may provide a significant advantage when performing tasks, such as the symmetry or XOR problem [24]. A great deal of research on integrated optical computing networks has been done using a cascaded Mach Zehnder interferometer (MZI) mesh [25–28]. MZI meshes have been widely used in linear optical circuits [25, 29], quantum information processing [30], universal multiport interferometers [27], optical modes descramblers [31, 32], and polarization processors [33]. For the linear section of optical neutral networks, impressive works, such as vowel recognition, have been demonstrated [34]. This method allows for good reconfigurability and independent control of both the amplitude and phase. However, the loading of the transmission matrix relies on iterative algorithms, which are quite slow and unsuitable for flexible matrix computations. Moreover, MZIs require a larger power consumption than resonant devices, such as microring resonators (MRRs), which are compact (several micron radius), more energy-efficient, highly integrated, and easily scalable [35, 36]. MRRs are resonant devices and the transmission coefficients are wavelength-sensitive. Parallel incoherent matrix computing can be achieved by controlling the resonant states of MRRs, which is commonly used in optical tensor computing and ONNs [11, 37]. The problem of MRR arrays is that the computation is incoherent, which means MRR arrays can only perform amplitude modulation without phase information. Thus, MMR arrays can only compute non-negative or real numbers assisted by differential detection. In addition, ultra-large-scale MRRs are difficult to implement because of the heavy thermal crosstalk and electronic circuits packaging. Hence, it is believed that MRRs cannot be implemented in a large-scale matrix multiplication to compute complex numbers.

In this paper, we present a systematic solution to extend the matrix computation of MRR arrays from the real-valued field to the complex-valued field, and from small scale (i.e., 4 × 4) to large scale matrix computation (i.e., 16 × 16). We experimentally demonstrate typical matrix–vector multiplication (MVM) applications of MRR arrays in Walsh Hardmard transform (WHT), discrete cosine transform (DCT), discrete Fourier transform (DFT), and image convolutional processing. These applications have significantly expanded the fields of optical computation based on MRR arrays. Our work shows huge potential for high-speed and universal matrix computations, such as applications in photonic accelerators and optical artificial intelligence.

## Principle

**Fig. 1:**
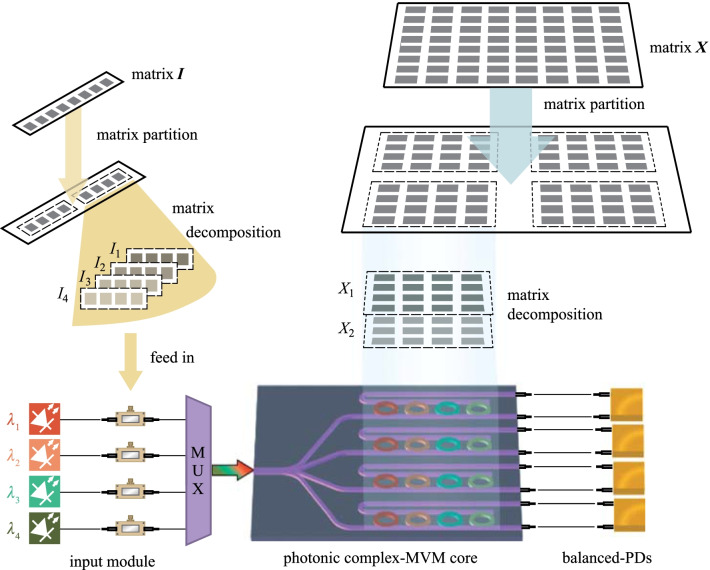
Working principle of complex-valued MVM. The entire architecture containing the input module, photonic complex-MVM core, and balanced-PDs. To realize the complex-valued MVM, the input matrix ***I*** and transmission matrix ***X*** are partitioned, decomposed, and subsequently fed into the input module and MVM core, respectively. The different colors correspond to different light wavelengths

The structure of the proposed on-chip MRR array (i.e., photonic complex-MVM core) is schematically illustrated in Fig. 1. The on-chip photonic complex-MVM core consists of a tunable silicon MRR array that includes 16 add-drop MRRs arranged in 4 rows and 4 columns. The entire architecture is based on wavelength-division multiplexing (WDM) and on-chip reconfigurable MRR array. The MRR array forms a complete network of a 4 × 4 transmission matrix, whose configuration can be realized by tuning the heater of each MRR.

Without consideration of the transmission loss, every add-drop MRR in each row of the array decides the through transmittance coefficient of $1-{a}_{ij}$ and drop transmittance coefficient of ${a}_{ij}$, respectively [38]. Then, the difference of these two ports is given by

1 $$\begin{array}{c} {\varvec{O}}= {\varvec{XI}}=\left[\begin{array}{cc}\begin{array}{cc}{1-2a}_{11}& 1-{2a}_{12}\\ 1-{2a}_{21}& 1-2{a}_{22}\end{array}& \begin{array}{cc}1-{2a}_{13}& 1-{2a}_{14}\\ 1-{2a}_{23}& 1-{2a}_{24}\end{array}\\ \begin{array}{cc}1-{2a}_{31}& 1-{2a}_{32}\\ {1-2a}_{41}& 1-{2a}_{42}\end{array}& \begin{array}{cc}1-{2a}_{33}& 1-{2a}_{34}\\ 1-{2a}_{43}& 1-{2a}_{44}\end{array}\end{array}\right]\lceil\begin{array}{c}\begin{array}{c}{i}_{1}\\ {i}_{2}\end{array}\\ \begin{array}{c}{i}_{3}\\ {i}_{4}\end{array}\end{array}\rceil,\end{array}$$

where the 4 × 1 vector ${\varvec{O}}={\left[{o}_{1}, {o}_{2}, {o}_{3}, {o}_{4}\right]}^{\mathrm{T}}$ represents the output vector, 4 × 1 vector ${\varvec{I}}={\left[{i}_{1}, {i}_{2}, {i}_{3}, {i}_{4}\right]}^{\mathrm{T}}$ represents the input vector, and 4 × 4 matrix ${\varvec{X}}$ stands for the transmission matrix. When the transmission loss is ignored, the drop port coefficient ${a}_{ij}$ falls in the range of $[0, 1]$ and the corresponding coefficient in the transmission matrix, defined by $1-{2a}_{ij}$, falls in the range of $[-1, 1]$. Thus, in the MVM operation, the input vector of ${\varvec{I}}$ is non-negative, while the transmission matrix of ${\varvec{X}}$ and the output vector of ${\varvec{O}}$ can cover the real number field.

Figure 1 also shows the working principle to extend the matrix computation of the MRR array from the real-valued field to the complex-valued field, and from small-scale (i.e., 4 × 4) to large-scale matrix computation. Combining matrix decomposition and matrix partition, our photonic complex-MVM chip can support arbitrary large-scale and complex-valued matrix computation.

Without loss of generality, the MVM consists of an 8 × 1 complex input matrix of ${\varvec{I}}$, 8 × 8 complex transmission matrix of ${\varvec{X}}$, and output matrix of ${\varvec{O}}$. To process a large amount of MVM, the size of the matrices is reduced through matrix partition. Matrix ${\varvec{I}}$ can be broken into two 4 × 1 matrices, while matrix ${\varvec{X}}$ can be divided into four 4 × 4 matrices. To process complex MVM in full complex number field, matrix ${\varvec{I}}$ is divided into ${{\varvec{I}}}_{1}$, ${{\varvec{I}}}_{2}$, ${{\varvec{I}}}_{3}$, ${{\varvec{I}}}_{4}$, defined as the positive real, positive imaginary, negative real, and negative imaginary parts of matrix ${\varvec{I}}$, respectively. Matrix ${\varvec{X}}$ is also divided into ${{\varvec{X}}}_{1}$ and ${{\varvec{X}}}_{2}$, representing the real and imaginary parts of ${\varvec{X}}$. The elements of the input submatrix, ${{\varvec{I}}}_{n}={\left[{i}_{1}, {i}_{2}, {i}_{3}, {i}_{4}\right]}_{n}^{\mathrm{T}} (n=1, 2, 3, 4)$, are loaded onto the beams with different wavelengths of ${\lambda }_{1}$, ${\lambda }_{2}$, ${\lambda }_{3}$, and ${\lambda }_{4}$ by optical intensity modulators (IMs). After mixing by a wavelength multiplexer (MUX), the input is equally divided into four branches, each of which consists of four independent MRRs aligned to resonate the ${\lambda }_{1}$, ${\lambda }_{2}$, ${\lambda }_{3}$, and ${\lambda }_{4}$ wavelengths, respectively. Matrix ${{\varvec{X}}}_{n} (n=1, 2)$ is loaded onto the photonic complex MVM core with the 4 × 4 MRR array, where the coefficients are determined by the voltages applied to each MRR. The output matrix of ${\varvec{O}}$ is detected by balanced photodetectors (PDs).

If the input vectors of ${{\varvec{I}}}_{1}$, ${{\varvec{I}}}_{2}$,…, ${{\varvec{I}}}_{m}$ are loaded in series, the input vector can be expanded into a *n* × *m* matrix where ${\varvec{I}}=[{{\varvec{I}}}_{1},\boldsymbol{ }{{\varvec{I}}}_{2},\boldsymbol{ }\dots ,\boldsymbol{ }{{\varvec{I}}}_{m}]$. Similarly, the corresponding output powers of ${{\varvec{O}}}_{1}$, ${{\varvec{O}}}_{2}$,…, ${{\varvec{O}}}_{m}$ should be measured in series so that the output *m* × *n* matrix can be written as ${\varvec{O}}=[{{\varvec{O}}}_{1},\boldsymbol{ }{{\varvec{O}}}_{2},\boldsymbol{ }\dots ,\boldsymbol{ }{{\varvec{O}}}_{m}]$. Hence, the MVM can be expanded into matrix–matrix multiplication denoted by the following equation:

2 $$\left[{\varvec{O}}_{1},{\varvec{O}}_{2},\dots,{\varvec{O}}_{m}\right]={\varvec{X}}\left[{\varvec{I}}_{1}, {\varvec{I}}_{2},\dots,{\varvec{I}}_{m}\right].$$

## Results

### Fabrication and experimental setup

The proposed device was fabricated on a silicon-on-insulator (SOI) platform. A 725 μm SOI wafer with 220 nm of top silicon and 2 μm of buried oxide (BOX) was used. The layout is transferred onto photoresist using electron beam lithography (EBL) and the top silicon is etched by inductively coupled plasma (ICP). The grating coupler is shallowly etched by 70 nm, while the silicon waveguide is fully etched by 220 nm. Between the waveguide and metal electrodes, 1 μm of silicon dioxide was deposited using plasma enhanced chemical vapor deposition (PECVD). The metal for the heaters and pads was deposited by electron beam evaporator (EBE). The heaters were made of 150 nm thick and 1 μm wide Ti. The electrical wires and pads were made of 20/250 μm thick Ti/Au.

**Fig. 2:**
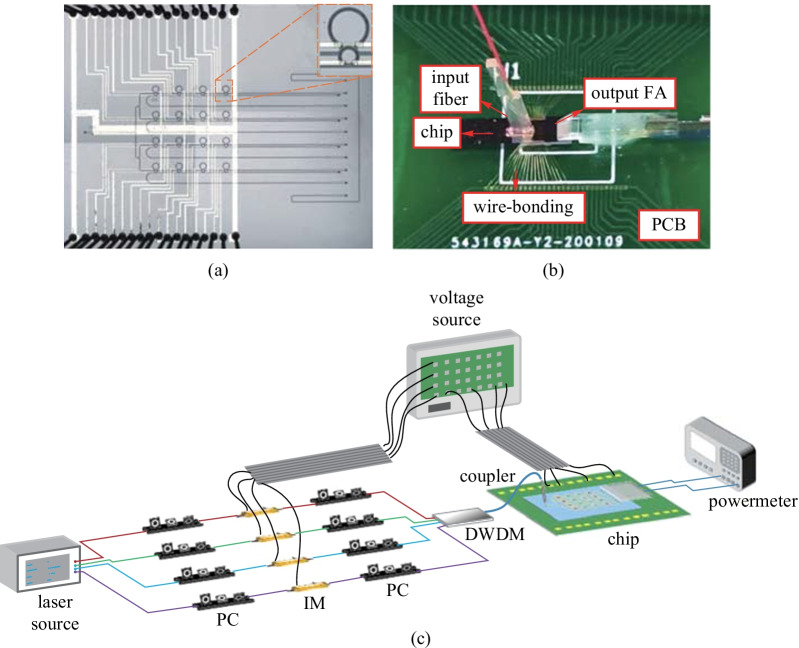
**a** Microscope image of the on-chip 4 × 4 MRR array, inset: zoomed in microscope image of the tunable MZI-MRR. **b** Image of the packaged chip. FA fiber array, PCB printed circuit board. **c **Experimental setup of the matrix arithmetic processor. PC polarization controller, IM intensity modulator, DWDM dense wavelength division multiplexing

The microscope image of the fabricated chip is illustrated in Fig. 2a. The input signal is injected through a grating coupler on the left and subsequently divided into four identical branches with a 4 × 4 MRR array. There are eight output gratings, representing the bus through waveguides and bus drop waveguides for each row of MRRs. The eight output gratings are placed in equal distances of 127 μm, the exact distance of the fiber array (FA) coupler. Figure 2b shows the packaged chip, where the metal pads are connected to the printed circuit board (PCB) by wire-bonding and the PCB is controlled by a custom 120-channel voltage source via a flexible flat cable. The input optical grating is coupled to an optical fiber that is vertically glued to the SOI chip. The output optical gratings on the chip are coupled to an optical FA that is attached to the PCB and equally distributed in 127 μm spacing V-groove, so that vertical output light from the chip is reflected 45° by the FA.

The experimental setup is shown in Fig. 2c. A continuous-wave (CW) laser was used as the stable optical source for the IMs. The electrical input data was encoded by a programmable voltage source and used as the driving signal that was temporally fed into the IMs. Since the output of the modulator is polarization-dependent, PCs were placed before and after the IMs to control the polarizations. A dense wavelength division multiplexing component (DWDM) was employed to combine the four wavelengths into a bus waveguide coupled to the packaged SOI chip. The optical powermeter is capable of both detecting and displaying the power values of the optical signals, which allowed us to obtain and record the results directly.

To verify the MVM function, IMs were used to configure the input vector ${\varvec{I}}$, while the transmission matrix ${\varvec{X}}$ was loaded by tuning the voltages applied on the MRR array. The output power values were then obtained from the balanced PDs. After calibration and normalization, the output vector ${\varvec{O}}$ was obtained. When the input is the identity matrix, the output matrix ${\varvec{O}}$ is equivalent to the transmission matrix ${\varvec{X}}$, allowing the transmission matrix ${\varvec{X}}$ to be directly read at the output ports. In practical situations, the variation ranges of through transmittance coefficient and drop transmittance coefficient are different due to MRR loss. In this case, the coefficients will require recalibration for actual optical matrix computation (See Appendix A).

To statistically describe the performance of this multiplier, over 500 sets of input vector data and matrix, ${\varvec{X}}$, were configured to the IMs and MRR array, respectively. Experimental results showed that the majority of the absolute values of the errors fall within the range of 0 − 0.1, which suggets rather accurate computing. See Appendix B for more details.

### Matrix–vector multiplication extending to the full real number field

Since the input vector ${\varvec{I}}$ was determined by the optical powers modulated by the IMs, the elements must be non-negative. Although the transmission matrix ${\varvec{X}}$ and output vector ${\varvec{O}}$ can only cover the real number field, our proposed scheme allows for the conversion of the input elements into negative values, extending the MVM to the full real number field.

**Fig. 3:**
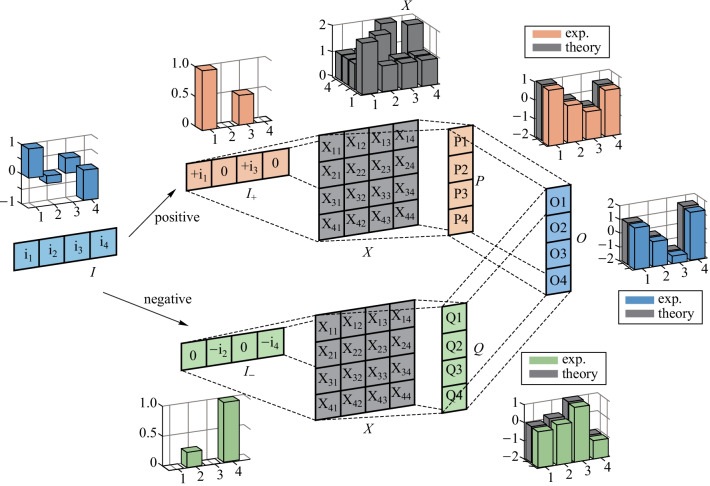
Matrix computation extending to the full real number field. The 4 × 1 block array represents the input or output vectors and the 4 × 4 block array represents the transmission matrix. The bar graph shows the results from one operation, where the inputs or experimental outputs are represented by the colored bars and the theoretical outputs or transmission matrix are represented by the gray bars

Figure 3 illustrates the proposed scheme. First, the input vector (real numbers) was divided into ${{\varvec{I}}}_{+}$, containing all the positive elements and zeros, and ${{\varvec{I}}}_{-}$, containing all the absolute values of the negative elements. The relationship between ${{\varvec{I}}}_{+}$, and ${{\varvec{I}}}_{-}$ are given by

3 $$\left\{\begin{array}{l}{{\varvec{I}}}_{+}=\frac{\left|{\varvec{I}}\right|+{\varvec{I}}}{2},\\{{\varvec{I}}}_{-}=\frac{\left|{\varvec{I}}\right|-{\varvec{I}}}{2},\\I={{\varvec{I}}}_{+}-{{\varvec{I}}}_{-}.\end{array}\right.$$

The resulting two non-negative vectors, ${{\varvec{I}}}_{+}$ and ${{\varvec{I}}}_{-}$, are subsequently used in place of the origin input vector. The transmission matrix ${\varvec{X}}$ was then loaded and the input vectors were configured as ${{\varvec{I}}}_{+}$ and ${{\varvec{I}}}_{-}$, respectively, to obtain the two output vectors, ${\varvec{P}}$ and ${\varvec{Q}}$. The targeted output matrix ${\varvec{O}}$ was obtained following subtraction operation. The relationships between ${\varvec{P}}$, ${\varvec{Q}}$, and ${\varvec{O}}$ are expressed below

4 $$\begin{array}{c}\left\{\begin{array}{c}P=X{{\varvec{I}}}_{+},\\ Q=X{{\varvec{I}}}_{-},\\ O=P-Q.\end{array}\right.\end{array}$$

Using the method described above, we were able to successfully split a real-valued optical MVM operation into two non-negative optical MVMs and one subtraction in the electrical domain. Figure 3 shows an experimental example of a real-valued MVM. The theoretical and experimental results are shown in three-dimensional bar graphs next to the corresponding matrices or vectors.

### Matrix–vector multiplication extending to the full complex number field

To further extend our matrix computation into the complex number field, the input vector ${\varvec{I}}$ and transmission matrix ${\varvec{X}}$ were both separated into a real part and imaginary part. The output vector can be expressed as

5 $$\begin{array}{c} {\varvec{O}}={\varvec{XI}}=\left(\mathrm{real}\left({\varvec{X}}\right)+\mathrm{i}*\mathrm{imag}\left({\varvec{X}}\right)\right)\left(\mathrm{real}\left({\varvec{I}}\right)+\mathrm{i}*\mathrm{imag}\left({\varvec{I}}\right)\right),\end{array}$$

where $\mathrm{i}$ is the square root of minus one, $\mathrm{real}({\varvec{M}})$ represents the real part of matrix ${\varvec{M}}$, and $\mathrm{imag}({\varvec{M}})$ represents the imaginary part of matrix ${\varvec{M}}\,(\mathrm{here}, {\varvec{M}}\,\mathrm{can\, be} \;{\varvec{X}}, \,{\varvec{I}}\, \boldsymbol{ }\mathrm{or}\boldsymbol{ } \, {\varvec{O}})$.

The matrix multiplication can then be divided into

6 $$\begin{array}{c}\left\{\begin{array}{c} {\rm real} \left({\varvec{O}}\right)={\rm real}\left({\varvec{X}}\right){\rm real}\left({\varvec{I}}\right)-{\rm imag}\left({\varvec{X}}\right){\rm imag}\left({\varvec{I}}\right),\\ {\rm imag}\left({\varvec{O}}\right)= {\rm real}\left({\varvec{X}}\right) {\rm imag} \left({\varvec{I}}\right)+ {\rm imag} \left({\varvec{X}}\right) {\rm real} \left({\varvec{I}}\right).\end{array}\right.\end{array}$$

**Fig. 4:**
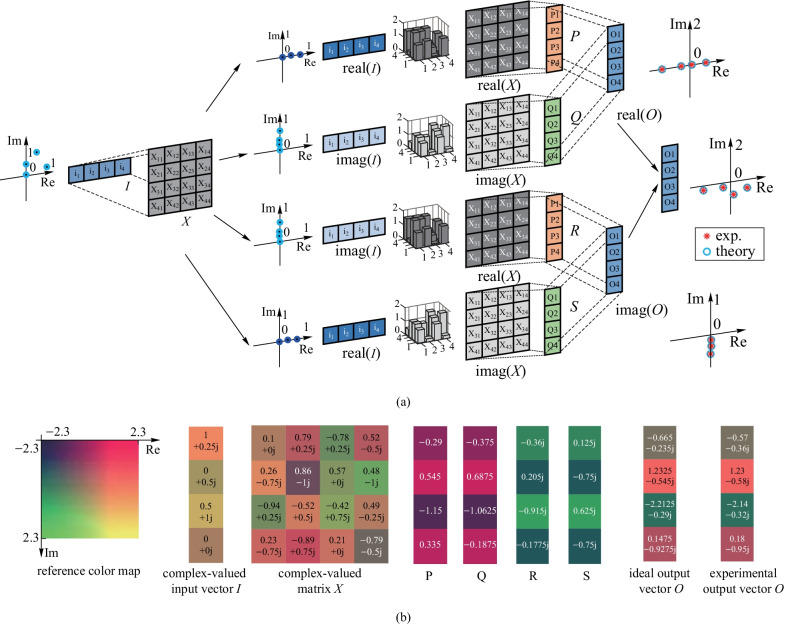
**a** Matrix computation extending to the full complex number field. The 4 × 1 block array represents the input or output vectors and the 4 × 4 block array represents the transmission matrix. The bar graph shows the theoretical transmission matrix. The coordinate figure shows the complex inputs or experimental complex outputs of the operation. **b** Theoretically expected and experimental results for the complex-valued matrix multiplication

As seen in Fig. 4a, the complex-valued matrix multiplication was divided into four operations of optical MVMs, specifically $\mathrm{real}\left({\varvec{X}}\right)\mathrm{real}\left({\varvec{I}}\right)$, $\mathrm{imag}\left({\varvec{X}}\right)\mathrm{imag}\left({\varvec{I}}\right)$, $\mathrm{real}\left({\varvec{X}}\right)\mathrm{imag}\left({\varvec{I}}\right)$, and $\mathrm{imag}\left({\varvec{X}}\right)\mathrm{real}\left({\varvec{I}}\right)$, as well as two operations of electrical addition or subtraction operations. Figure 4a also shows an experimental demonstration of complex MVM. The two-dimensional coordinate diagrams in blue dots represent the corresponding input vectors or output vectors, and the three-dimensional gray bar graphs represent the transmission matrix. The experimental results are consistent with the theoretical results. In addition, the experimental results presented in Fig. 4b of the output of complex-valued matrix multiplication are also consistent with the predicted results.

### Matrix–vector multiplication extending to higher dimensions

**Fig. 5:**
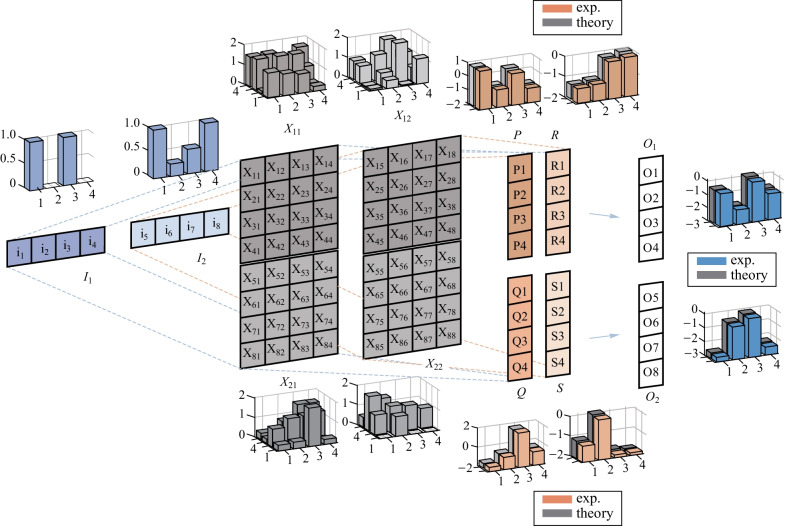
Example of the partition of an 8 × 8 MVM. The 4 × 1 block array represents the input or output vectors, and the 4 × 4 block array represents the transmission matrix. The bar graph shows the results from one operation, where the inputs or experimental outputs are represented by colored bars and the theoretical outputs are represented by gray bars

Considering the fact that partition of matrix can enlarge the matrix dimension, we were able to implement a high dimensional MVM with low dimensional MRR array via matrix partition. Figure 5 illustrates the basic principle of matrix partition. The input and output data are 8 × 1 vectors and the transmission matrix of ${\varvec{X}}$ is an 8 × 8 matrix. To execute the 8 × 8 matrix computation using our 4 × 4 processor, the input and output vectors have to be split into two 4 × 1 vectors. Meanwhile, the transmission matrix is broken into four 4 × 4 matrices. Therefore, the equation can be written as

7 $$\begin{array}{c} {\varvec {O}}=\left(\genfrac{}{}{0pt}{}{{{\varvec{O}}}_{1}}{{{\varvec{O}}}_{2}}\right)=\left(\begin{array}{cc}{{\varvec{X}}}_{11}& {{\varvec{X}}}_{12}\\ {{\varvec{X}}}_{21}& {{\varvec{X}}}_{22}\end{array}\right)\left(\genfrac{}{}{0pt}{}{{{\varvec{I}}}_{1}}{{{\varvec{I}}}_{2}}\right)=\left(\genfrac{}{}{0pt}{}{{{\varvec{X}}}_{11}{{\varvec{I}}}_{1}+{{\varvec{X}}}_{12}{{\varvec{I}}}_{2}}{{{\varvec{X}}}_{21}{{\varvec{I}}}_{1}+{{\varvec{X}}}_{22}{{\varvec{I}}}_{2}}\right).\end{array}\boldsymbol{ }$$

Therefore, the partition of matrix can be realized by four rounds of optical MVMs and two rounds of electrical additions. Figure 5 shows an experimental demonstration of a partition of MVM, where the theoretical or experimental results are given in the three-dimensional bar graphs. It can be also seen from Fig. 5 that the experimental results are in agreement with the theoretical predictions.

### Applications in signal transformation and image processing

**Fig. 6:**
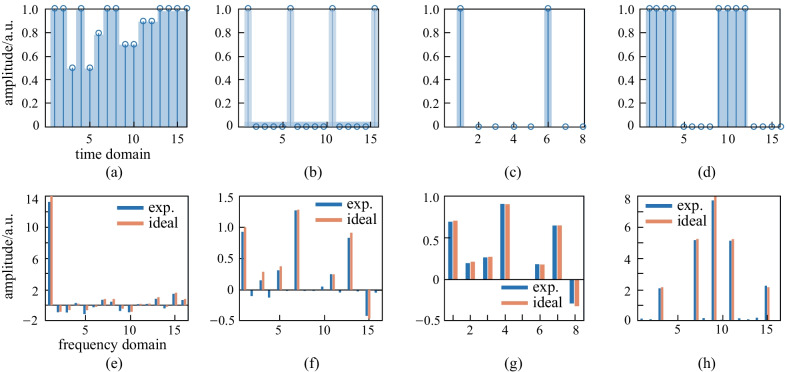
Input and output signal sequence of three signal transformation. The input sequence of **a** WHT, **b** and **c**, DCT, and **d** DFT. **e** Experimental results (blue bars) and ideal (red bars) output of WHT. **f** and **g** Experimental results (blue bars) and ideal (red bars) output of DCT. **h** Experimental results (blue bars) and ideal (red bars) output of DFT

Modern signal and image processing are two fields where algorithms based on large complex MVMs are widely utilized. This paper demonstrates three typical signal transformations, specifically, discrete WHT, DCT, and DFT [39]. WHT is orthogonal transformation that is widely used in imaging and code division multiple access [40]. The Hadamard matrix elements are equal to 1 or − 1, so that there are only addition and subtraction operations in the calculation, making it much simpler than DFT and DCT. Energy concentration is a characteristic of WHT, meaning the more uniform the numbers in the original data are, the more concentrated the transformed data are on the side. This property makes WHT advantageous for image compression [41]. Figure 6a shows the input signal and Fig. 6e shows the transformed signals after our matrix size to 16 × 16 was extended. One can see that WHT can compress information in the low frequency region if the input signal has a uniform amplitude distribution, thus the high frequency region can be ignored since it has a very low amplitude. DCT plays an important role in signal processing, signal modulation, and demodulation [42]. A periodic sequence was input into a 16 × 16 network and the output matrix was calculated, as shown in Fig. 6b and f. The first half of the former sequence was loaded into an 8 × 8 network as the input, depicted in Fig. 6c. The resulting output vector is quite similar to that presented in Fig. 6f and g. These results reflect the symmetry of DCT and provide supporting evidence that our system can correctly perform DCT. In addition, DFT can convert a signal sampling in time domain into frequency domain, one of the most frequently used operations in signal transformation [43]. Here, we used an input signal in the form of a square wave. Since DFT is a complex transformation, the amplitude of the output sequence is shown in form of its absolute value, which is shaped in a sinc function, as shown in Fig. 6d and h. The results show that not only can DFT be performed by our system, the calculation errors are also very small.

**Fig. 7:**
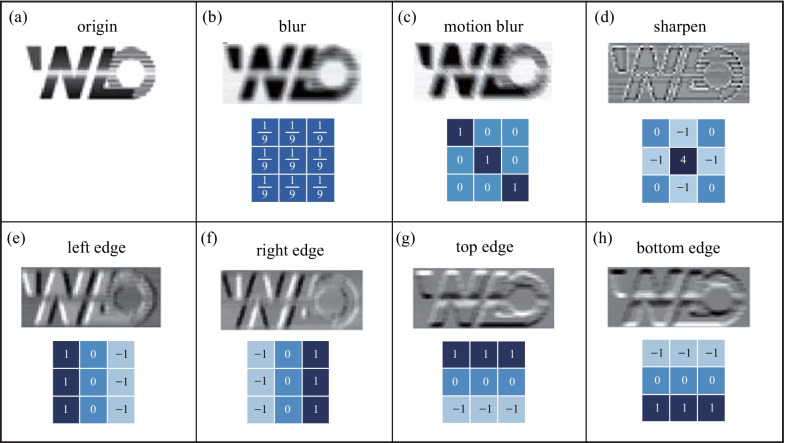
Experimental results of image convolution. **a** Original image, which is the logo of Wuhan National Laboratory for Optoelectronics (WNLO). **b**−**h** Convolved images and the corresponding transmission matrices of 3 × 3 kernels. The kernels are designed to perform different image processing functions or highlight different edges of the original image

Image convolution is of paramount importance to convolutional neural networks and image processing, which can be performed in optical domain to achieve convolutional acceleration. To experimentally verify image convolution with our MVM, we choose the logo of Wuhan National Laboratory for Optoelectronics (WNLO) as an example, as well as seven different 3 × 3 sized kernels. The kernels are designed to perform different image processing functions or highlight different edges of the original image. The pixel values of the input image are loaded into the IMs by the electrical waveform and the on-chip MRR array is loaded by the transmission matrix representing the kernel. Figure 7 shows the experimental results, including the recovered feature maps and corresponding transmission matrices of the kernels.

Compared with the original image, the edge features of the processed image are clearly visible in Fig. 7e−h, demonstrating the effectiveness of the optical convolution operation. The kernels in Fig. 7b–d correctly performed different image processing functions, including blur, motion blur, and sharpen. The kernels in Fig. 7e−h highlighted the edges of the original image in different directions. Using the theoretical results as reference, we determined that the calculation errors of the optical convolution operation was mainly concentrated on the bright part (i.e., high pixel value area) of the image, which indicates that these errors are largely caused by thermal crosstalk, rather than noise. Real-time calibration algorithms and external temperature control devices are implemented for system stability.

## Discussion and future perspective

The experimental results of both signal and image processing clearly demonstrate that our proposed system is able to extend matrix computation to (1) real numbers, (2) full complex numbers, (3) higher processing dimensions, and (4) convolution. Thus, the processor can serve as a universal matrix arithmetic processor for complex tasks in various application scenarios.

**Fig. 8:**
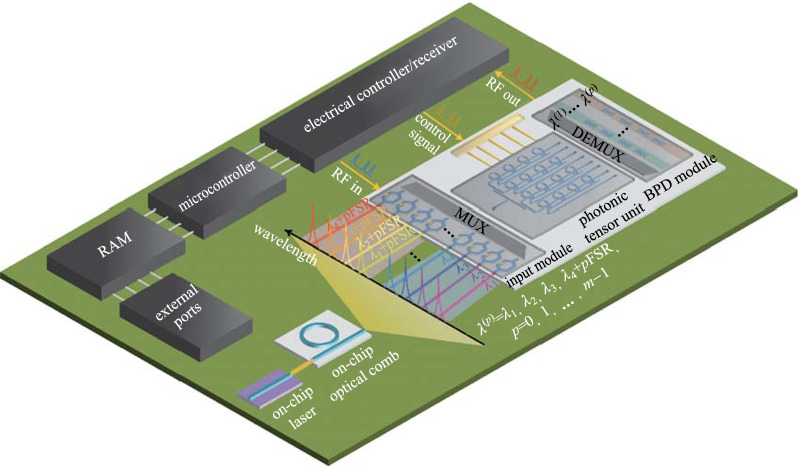
Highly integrated on-chip scheme for optical parallel computation. There are *m* sets of different input vectors provided by multiwavelength light source (e.g., on-chip optical comb). Input signals are modulated in different wavelengths by the IMs, then multiplexed as the input of MRR array via wavelength division multiplexers (MUXs). The output powers of each row in MRR array are divided by the wavelength division demultiplexers (DEMUXs) and separately detected by m sets of corresponding photodiodes. Each set of wavelengths is used for one input vector. The electrical controller/receiver are driven by microcontroller equipped with RAM and external ports

However, the processor can be further improved in several ways. For example, the computational efficiency can be multiplied by making full use of parallel computation or by increasing the number of input wavelengths. Note that the transmission spectrum of MRR is repeated with a period of about 6 nm, which represents the free spectral range (FSR) of MRR. Therefore, multiple sets of input vectors with an interval equal to FSR can be operated simultaneously, as shown in Fig. 8. Suppose that there are m sets of different input vectors and the wavelengths of the input matrices are set as $\left({\lambda }_{1}, {\lambda }_{2}, {\lambda }_{3}, {\lambda }_{4}\right)+p\mathrm{FSR}$, where $p=0, 1,\dots , m-1$. To obtain the output data, the output powers of each row are divided by the wavelength-division multiplexer and separately detected by m sets of corresponding balanced PDs. In this process, the state of the transmission matrix is fixed (i.e., the state of MRR array is fixed), while the m sets of input and output vectors are independently paralleled. This means that m sets of MVMs can be executed simultaneously, demonstrating the possibility of parallel optical computation. Secondly, full integration is crucial to improve the competitiveness of optical computing compared to electrical matrix processing. As shown in Fig. 8, an optical comb is integrated into the chip, providing a series of comb lines that are modulated by IMs of the input module. With this, the experimental setup is greatly simplified. The thermally tuned MRRs can be replaced by electrically tuned ones, which might improve the response rate by several orders of magnitude. As for electrical control, the electrical controller/receiver, together with microcontroller, random access memory (RAM), and external ports are applied to improve system response rate.

## Conclusion

In conclusion, we have demonstrated a small MRR array that performs large complex MVM. Through matrix decomposition and partition, we have also optimized the photonic complex-MVM core so that it can perform larger complex MVM and extended its matrix computation to (1) real number, (2) complex number, and (3) higher processing dimensions. We have fabricated the integrated photonic complex-MVM core on an SOI platform, which is compact and compatible with CMOS technology. With a small MRR array, the 4 × 4 matrix computation system can be scaled up to 8 × 8, 16 × 16, or even larger operation dimensions in complex field with traditional incoherent computing. The processor was then applied for WHT, DCT and DFT signal transformations. Image processing with 7 types of convolutional kernels is also experimentally demonstrated. Our proposed system shows adequate performance in various applications. The processing capacity of this matrix–vector multiplier can be further enhanced by enabling parallel WDM computation and full integration with on-chip laser sources and electrical microcontrollers in the future.

## Figures and Tables

**Fig. 9:**
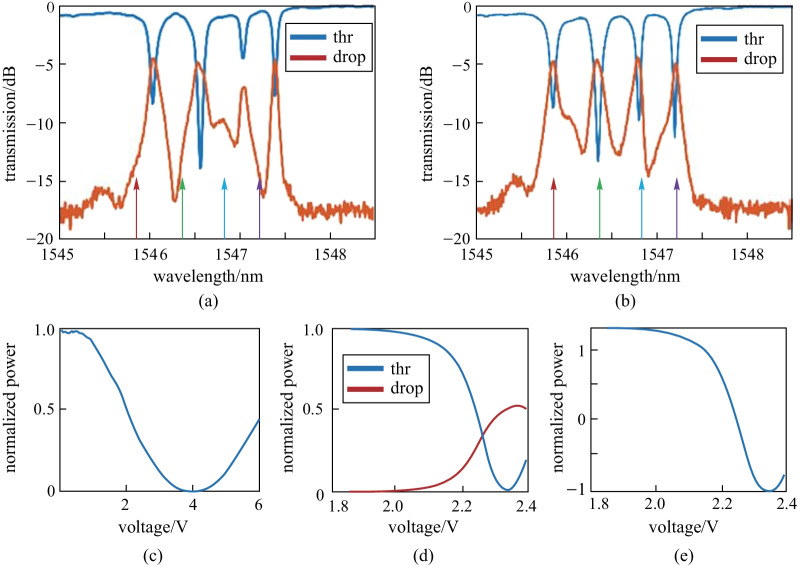
Calibration of MRR array. **a** and **b** are the spectra before and after laser wavelength calibration respectively, and the four laser wavelengths are represented by four colors (red, green, blue, purple) respectively. **c** Voltage-input relational table. **d** Normalized optical power of the through port (red line) and drop port (blue line) of a certain MRR. **e** Normalized optical power curve after difference calculation

**Fig. 10:**
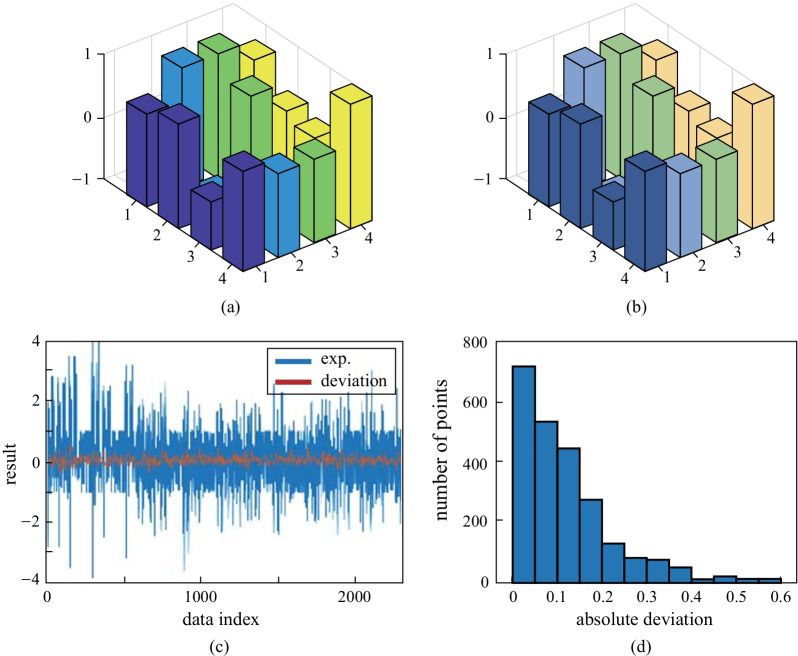
Experimental results of matrix–vector multiplication. **a** Sample experimental transmission function of ${\varvec{X}}$ and **b** its corresponding theoretical results. **c** Result vector data and **d** error distribution

## Appendix

### A. Calibration of MRR array

Since the MRR is a resonant device, the transmittance of the through and drop ports depends on the difference between the laser and resonance wavelength of the MRR. Therefore, the four laser wavelengths need to be calibrated at the resonance peak of the corresponding MRR prior to experimentation. Figure 9a shows the state in which the laser wavelength is not aligned with the resonant peak of the MRR. In this case, the transmission coefficient of the MRR is ${x}_{ij}=1$. As shown in Fig. 9b, the voltage values of the four MRRs were changed so that the four laser wavelengths coincide with the resonant peak of the MRRs, where the transmission coefficients were all* x*_*ij*_ = 1. The calibration of the ring array is between these two states. Figure 9c shows the normalized power detected at the through port when the MRR is fixed in the all-pass state (i.e., the transmission coefficient of the MRR is 1) and the voltage applied on the IM is changed. The voltage-input relational table was obtained by choosing a fixed step length of 20 mV, applying 300 V steps to the IM, and measuring the corresponding output power. When a particular input needs to be loaded, the computer applies table look-up and loads the corresponding voltage into the IM. Similarly, the table look-up method is used in MRR calibration. First, the corresponding input is set at the maximum value of 1 and the voltages are selected according to a fixed step size between ${x}_{ij}=-1$ and ${x}_{ij}=1$. Then, the voltages are applied to the MRR array and the output powers of MRR are measured. The voltage-transmission relational table was obtained and shown in Fig. 9d and e. When a particular transfer coefficient need to be loaded, the computer looks up the nearest value in the table using the look-up table method and loads the corresponding voltage onto the MRR electrodes.

### B Experimental verification of matrix–vector multiplier

Figure 10a presents a sample experimental transmission function of ${\varvec{X}}$, Fig. 10b lists the corresponding theoretical results, and Fig. 10c summarizes the vector data results. Each data point represents a dot product of one of the row vectors of ${\varvec{X}}$ and the input vector. The blue line represents the experimental results and the red line represents the deviation of each experimental point. The error statistics are calculated and shown in Fig. 10d, where most of the absolute values of the errors fall within the range of 0–0.1.
